# Effect of Maternal Age on Pregnancy or Neonatal Outcomes Among 4,958 Infertile Women Using a Freeze-All Strategy

**DOI:** 10.3389/fmed.2019.00316

**Published:** 2020-01-10

**Authors:** Jiaying Lin, Jialyu Huang, Qianqian Zhu, Yanping Kuang, Renfei Cai, Yun Wang

**Affiliations:** Department of Assisted Reproduction, Shanghai Ninth People's Hospital, Shanghai Jiao Tong University School of Medicine, Shanghai, China

**Keywords:** advanced reproductive age, frozen–thawed embryo transfer, freeze-all strategy, pregnancy outcome, neonatal outcome

## Abstract

The freeze-all strategy has been increasingly employed in the context of *in vitro* fertilization (IVF) cycles globally, but the relative advantages of this approach are not entirely understood. Herein we sought to assess how maternal age affected pregnancy and neonatal outcomes in women who had undergone frozen–thawed embryo transfer (FET). In this retrospective analysis, we assessed outcomes for 4,958 total women at the University-affiliated Tertiary Centre from January—December 2017. We compared pregnancy and neonatal outcomes between a control group (<30 years old) and groups of more advanced maternal age (30–34, 35–37, 38–40, 41–43, and 44–50 years). We found that live birth rates (LBR) for the first FET cycle following a freeze-all strategy significantly declined with increasing maternal age, with the most pronounced declines in the 35–37 and 38–40 age groups (LBR: 51.12% at <30 years, 43.86% at 30–34 years, 41.64% at 35–37 years, 25.67% at 38–40 years, 15.58% at 40–43 years, and 4.78% at 44–50 years, respectively). Rates of preterm delivery (PTD), very PTD, low birth weight (LBW), very LBW, term LBW, preterm LBW, and macrosomia were comparable across study groups. Together these results thus suggest that increasing maternal age has an adverse impact on pregnancy outcomes without affecting PTD or LBW risk in the context of a freeze-all strategy.

## Introduction

As cryopreservation methods have become more standardized and reliable, preserving embryos in this fashion has become an increasingly common strategy in the context of *in vitro* fertilization (IVF), with many facilities implementing a freeze-all policy (1). For such a strategy, all embryos are frozen before being transferred into the mother during a later naturally or medically-induced cycle. Following frozen-embryo transfer (FET), some studies have suggested that singleton pregnancies are more likely to result in a low birth rate (LBR) relative to fresh-embryo transfer strategies (2). However, in a meta-analysis integrating findings from 11 observational studies, it was determined that FET was overall associated with reductions in the risk of low birth weight (LBW), preterm birth (PTB), and perinatal death than were pregnancies resulting from fresh-embryo transfer (3, 4).

Assisted reproductive technologies (ARTs) are most often utilized by women in their mid-30s, but the increasing availability of contraception and other changing trends with respect to professional and personal expectations have led to increasing delays in the age at which women most often seek to have children (5). This has led to an increasing demand for the use of ART among women who are of a more advanced reproductive age, with the average age of women bearing children having increased globally from the early 20s to early 30s in recent decades (6). Such increasing maternal age is well known to be a risk factor for higher rates of negative maternal and neonatal outcomes, including PTB, LBW, and small-for-gestational age (SGA) in women who have conceived spontaneously (7–9). Less well understood, however, is how age influences the risk of such outcomes in ART-derived pregnancies (10, 11). Studies completed to date have largely assessed outcomes for fresh transfer cycles (10, 11), and have not assessed whether the high estrogen levels present in the context of COH could have a negative impact on the endometrium (12). As such, it is possible that FET-derived pregnancy outcomes may differ significantly from those resulting from fresh-embryo transfer owing to the more physiological endometrial environment, potentially resulting in better maternal and neonatal outcomes (13, 14).

Therefore, this study aimed to conduct a comprehensive analysis of how maternal age affects pregnancy and neonatal outcomes for singleton births resulting from a freeze-all FET strategy.

## Materials and Methods

This study was retrospective and non-interventional in nature, with women who underwent their first freeze-all strategy-based FET cycle from 1 January 2017 to 31 December 2017 at the Department of Assisted Reproduction of Shanghai Ninth People's Hospital, Shanghai Jiaotong University School of Medicine being enrolled. The application of a freeze-all strategy was decided before initiating ovarian stimulation. The Institutional Review Board of the Ninth People's Hospital of Shanghai approved this study. This study was carried out in accordance with the Helsinki Declaration. Due to the retrospective nature of this analysis, informed consent was not required and patient data was used anonymously.

## Inclusion Criteria

Women from 22–50 years of age at time of FET were enrolled in the study, and were grouped based on age: Group 1 (G1 = 1,248 patients; <30 years old), Group 2 (G2 = 1,831 patients; 30–34 years old), Group 3 (G3 = 819 patients; 35–37 years old), Group 4 (G4 = 522 cycles; 38–40 years old), Group 5 (G4 = 308 cycles; 41–43 years old), and Group 6 (G4 = 230 cycles; 44–50 years old). Group demographic characteristics are compiled in Table 1.

**Table 1:** Baseline characteristics of all FET cycles.

**Characteristic**	**<30 y (*N* = 1,248)**	**30–34 (*N* = 1,831)**	**35–37 (*N* = 819)**	**38–40 (*N* = 522)**	**41–43 (*N* = 308)**	**44–50 (*N* = 230)**
Age of embryo transfer, mean (SD), years	27.32 ± 1.42	32.11 ± 1.42	35.95 ± 0.93	38.99 ± 0.86	41.93 ± 0.88	46.89 ± 1.96
Duration of infertility (years)	2.21 ± 1.75	3.19 ± 1.89	3.69 ± 2.37	4.39 ± 2.88	5.36 ± 3.83	6.11 ± 4.76
BMI of women	23.09 ± 4.36	22.87 ± 4.67	23.18 ± 4.53	23.36 ± 4.64	23.63 ± 4.33	23.53 ± 4.73
Antral follicle count (*n*)	12.53 ± 8.19	10.68 ± 7.79	8.89 ± 7.49	7.49 ± 7.43	6.06 ± 6.22	4.39 ± 4.67
**Infertility causes**, ***n***						
Tubal infertility	489 (39.19%)	740 (40.41%)	367 (44.75%)	243 (46.53%)	144 (46.89%)	106 (45.87%)
Anovulatory	117 (9.38%)	196 (10.69%)	21 (2.62%)	12 (2.39%)	6 (1.86%)	4 (1.83%)
Endometriosis	102 (8.14%)	172 (9.39%)	44 (5.43%)	28 (5.39%)	17 (5.49%)	10 (4.21%)
Male cause	195 (15.66%)	170 (9.28%)	85 (10.39%)	46 (8.82%)	26 (8.49%)	17 (7.18%)
Unexplained infertility	30 (2.44%)	43 (2.36%)	21 (2.52%)	10 (1.92%)	15 (4.93%)	14 (6.09%)
Combined	315 (25.19%)	510 (27.87%)	281 (34.29%)	183 (34.95%)	100 (32.34%)	79 (34.82%)
Previous IVF failures, *n*						
0	1,093 (87.55%)	1,661 (90.72%)	700 (85.47%)	455 (87.16%)	256 (83.12%)	190 (82.61%)
1–2	134 (10.74%)	105 (5.73%)	75 (9.16%)	41 (7.85%)	32 (10.39%)	23 (10.00%)
≥3	21 (1.71%)	65 (3.55%)	44 (5.37%)	26 (4.99%)	20 (6.49%)	17 (7.39%)
**Type of infertility**						
Primary	843 (67.57%)	1,086 (59.31%)	458 (55.87%)	246 (47.18%)	120 (39.01%)	57 (24.81%)
Secondary	405 (32.43%)	745 (40.69%)	361 (44.13%)	276 (52.82%)	188 (60.99%)	173 (75.19%)
**Type of FET cycle**						
Natural	241 (19.32%)	379 (20.72%)	217 (26.53%)	147 (28.12%)	91 (29.39%)	68 (29.53%)
HRT	320 (25.63%)	522 (28.53%)	260 (31.79%)	173 (33.23%)	117 (37.83%)	104 (45.21%)
Ovarian stimulation	687 (55.05%)	930 (50.75%)	342 (41.68%)	202 (38.65%)	100 (32.78%)	58 (25.26%)
**Endometrial thickness**						
**Fertilization method**						
IVF	730 (58.53%)	1,142 (62.39%)	532 (64.98%)	347 (66.42%)	204 (66.29%)	70 (30.56%)
ICSI	371 (29.69%)	472 (25.76%)	190 (23.23%)	128 (24.53%)	86 (27.93%)	154 (66.92%)
Half IVF + half ICSI	147 (11.78%)	217 (11.85%)	97 (11.79%)	47 (9.05%)	18 (5.78%)	6 (2.52%)

## Ovarian Stimulation

Patients in this study underwent either gonadotropin-releasing hormone antagonist (GnRH-ant) treatment, mild stimulation, or progestin-primed ovarian stimulation (PPOS), as discussed in detail in past reports (15, 16). For the GnRH-ant protocol, participants received daily human menopausal gonadotropin (hMG; 150–225 IU; Anhui Fengyuan Pharmaceutical Co., China) from MC3, and once the largest follicle reached a size of >12–14 mm they received 0.25 mg GnRH-ant (Cetrorelix, Mercerono) daily until the trigger day. In patients that underwent mild stimulation, patients received clomiphene citrate (CC, Fertilan, Codal-Synto Ltd, 25 mg/day) and letrozole (LE, Jiangsu Hengrui Medicine Co., 2.5 mg/day) from MC3 onwards, but LE was given for only 4 days, and CC was continuously administered until the trigger day. Patients were injected with 150 IU hMG every other day, beginning on MC6. Patients stimulated via the PPOS regimen received 10 mg/day medroxyprogesterone acetate (Shanghai Xinyi Pharmaceutical Co., China) along with hMG (150–225 IU) per day from MC3 to trigger day. A transvaginal ultrasound examination was performed 5 days later to record the number of developing follicles, and serum hormone concentrations were measured. The hMG doses were adjusted according to ovarian response, which was assessed based upon serum estradiol (E2) levels and transvaginal ultrasound (TVU) results. Once three or more follicles were 18 mm or larger in diameter, or one follicle was 20 mm or larger in diameter, patients were given 1,000–5,000 IU human chorionic gonadotropin (hCG; Lizhu Pharmaceutical Trading Co., China) along with 0.1–0.2 mg triptorelin as a means of inducing final oocyte maturation.

In all cases, 34–36 h after administering the trigger, oocytes were retrieved and fertilized via conventional IVF/ICSI approaches based on semen sample characteristics. Zygotes were then cultured via Continuous Single Culture (Irvine Scientific, USA) for 3 days, after which Cummin's criteria were used for embryonic grading (17), with those of good quality (grade I–II) being chosen for vitrification. Lower quality (grade III–IV) embryos were cultured for a longer period of time, and the Gardner and Schoolcraft scoring criteria (18) were then used to identify good quality blastocysts (grade ≥3 BC), which underwent vitrification on days 5–6. All vitrification and thawing was conducted as described previously (15).

## Endometrial Preparation and FET

FET cycle endometrial preparation was conducted based either upon a natural cycle, mild ovarian stimulation, or hormone replacement therapy as in previous reports (19). In natural cycles, serum hormones and ultrasound imaging were used to monitor follicular growth beginning on day 10 of the cycle. Once appropriate parameters were detectable (endometrial thickness >8 mm; dominant follicle diameter > 16 mm, E2 > 150 pg/mL, *P* < 1.0 ng/mL) luteinizing hormone (LH) levels were assessed and patients were either given 5,000 IU hCG at night (21:00) if LH levels were <20 IU/L in order to induce ovulation, with 3-day-old embryos being transferred after 5 days, or they were given 5,000 IU hCG in that same afternoon if LH levels were >20 IU/L, with embryonic transfer 4 days later. Similarly, blastocyst transfer was conducted after 6 or 7 days based on hormone levels and ultrasound findings. Three days following hCG administration, patients were given dydrogesterone (40 mg/day) (Duphaston; Abbott Biologicals B.V., USA) for luteal phase support. In patients exhibiting irregular menstruation following ovarian stimulation, 2.5–5 mg letrozole was given from cycle days 3–7 to promote mono-follicular growth, monitoring follicle growth starting on day 10. Where appropriate, women were given hMG (75 IU per day) to promote growth of the follicle and the endometrial lining. These criteria guided hCG (5,000 IU) and FET timing. In patients exhibiting a thin endometrium in the context of natural or stimulated cycles, oral E2 (0.025 mg/day) (Shanghai Xinyi Pharma) was given starting on day 3 of the cycle to induce appropriate endometrial preparation. Once the endometrium achieved a >8 mm thickness, patients were further given Femoston (8 mg/day) (Solvay Pharmaceuticals B.V., Brussels, Belgium) for 3 days, after which embryonic transfer was conducted. After pregnancy was successfully induced in patients, exogenous estrogen and progesterone supplementation was conducted for 8–10 weeks.

## Pregnancy Outcomes

Measurements of serum β-hCG and ultrasonography were used to monitor pregnancy outcomes such as implantation rate, clinical pregnancy rate, and LBR for a given transfer cycle. Implantation rates were determined according to how many gestational sacs were detectable via ultrasonographic assessment relative to the total number of embryos transferred. Clinical pregnancy rates were determined based upon ultrasonographic evidence of a gestational sac within the uterus 6–8 weeks following transfer, with the overall rate being determined based on the number of pregnancies divided by the number of FET cycles. Live births were those in which infants exhibited signs of life following delivery at least 24 weeks post-gestation, with LBR determined based on live births per FET cycle. Rates of negative outcomes such as miscarriage and ectopic pregnancies were also assessed.

We further assessed rates of PTD (delivery before gestational week 37), LBW (birth weight < 2,500 g), term LBW (LBW in neonates born at least 37 weeks post-gestation), preterm LBW (LBW in pre-term neonates), very PTD (delivery before gestational week 32), very LBW (birth weight < 1,500 g), and macrosomia (birth weight > 4,000 g).

## Statistical Analysis

Data are given as means with standard deviations and percentages (20). Continuous data were compared via student's *t*-tests, while chi-squared tests were used to compare proportions. The influence of maternal age on pregnancy outcomes was assessed via a regression model. Where appropriate, odds ratios (OR) and corresponding 95% confidence intervals (CIs) were calculated, using women <30 years old as a reference. *P* < 0.05 was the significance threshold. SPSS v16.0 (SPSS Inc., IL, USA) was used for all analyses.

## Results

### Patient Characteristics

Patient baseline characteristics are compiled in Table 1, with patient age having been used to define six study groups (<Age 30, 25.17%; Age 30–34, 36.93%; Age 35–37, 16.52%; Age 38–40, 10.53%; Age 41–43, 6.21%; Age 44–50, 4.64%). Reductions in primary infertility and antral follicle count were evident with increasing age, whereas rising maternal age was associated with a longer duration of infertility. In all age groups, tubal defects were a primary driver of infertility. There were no significant differences in maternal BMI, mean LH at baseline, mean *P*, FET cycle type, or fertilization method used between groups.

### Pregnancy Outcomes

All embryos in the present study underwent vitrification, with a 99.60% survival rate upon thawing and warming. Pregnancy outcomes after embryo transfer are compiled in Table 2. We observed a significant increase in miscarriage rate with increasing maternal age, whereas implantation, clinical pregnancy, multiple pregnancy, and live birth rates significantly declined with increasing age (Table 2). The greatest declines were evident in the 35–37 and 38–40 year old groups (LBR: 51.12% at <30 years, 43.86% at 30–34 years, 41.64% at 35–37 years, 25.67% at 38–40 years, 15.58% at 40–43 years, and 4.78% at 44–50 years). Ectopic pregnancy rates were comparable among groups, with the exception of a slightly higher rate in the <30 year old group relative to the 44–50 year old group (4.02 vs. 0%).

**Table 2:** Reproductive outcomes biy age group.

**Characteristic**	**<30 y** **(*N* = 1248)**	**30–34** **(*N* = 1831)**	**35–37** **(*N* = 819)**	**38–40** **(*N* = 522)**	**41–43** **(*N* = 308)**	**44–50** **(*N* = 230)**	***P***
Number of frozen embryos	2,278	3,277	1,418	885	514	383	
Number of viable embryos after thawed	2,272	3,262	1,411	879	514	382	
**Embryo quality**							
High-quality embryos	2,262 (99.59%)	3,240 (99.33%)	1,403 (99.45%)	870 (99.02%)	509 (98.98%)	375 (98.04%)	a, b, c, d, e^*^
Low-quality embryos	10 (0.41%)	22 (0.67%)	8 (0.55%)	9 (0.98%)	5 (0.97%)	7 (1.96%)	
**Number of embryo transferred**							
1	342	488	201	143	86	70	a, b, c, d, e
2	1,930	2,774	1,210	736	428	312	
**Embryo developmental stage at transfer**							
Cleavage stage embryos	2,085	3,015	1,291	808	460	365	a, b, c, d, e^*^
Blastocyst stage embryo	187	247	120	71	54	17	
Clinical pregnancy rate per transfer	56.09% (700/1,248)	49.97% (915/1,831)	47.99% (393/819)	36.78% (192/522)	25.00% (77/308)	12.17% (28/230)	a^*^, b^*^, c^*^, d^*^, e^*^
Implantation rate	40.63% (923/2,272)	35.74% (1,166/3,262)	33.09% (467/1,411)	25.03% (220/879)	15.37% (79/514)	7.59% (29/382)	a^*^, b^*^, c^*^, d^*^, e^*^
Miscarriage rate	8.43% (59/700)	11.48% (105/915)	12.21% (48/393)	29.69% (57/192)	36.36% (28/77)	64.29% (18/28)	a^*^, b^*^, c^*^, d^*^, e^*^
Multiple pregnancy rate	31.86% (223/700)	27.43% (251/915)	18.82% (74/393)	14.58% (28/192)	2.59% (2/77)	3.57% (1/28)	a, b^*^, c^*^, d^*^, e^*^
Ectopic pregnancy rate	4.02% (29/722)	3.08% (29/942)	1.51% (6/399)	3.52% (7/199)	2.53% (2/79)	0% (0/28)	a, b, c, d, e^*^
Live birth rate	51.12% (638/1,248)	43.86% (803/1,831)	41.64% (341/819)	25.67% (134/522)	15.58% (48/308)	4.78% (11/230)	a^*^, b^*^, c^*^, d^*^, e^*^

*a:<30 y vs. 30–34 y*.

*b: 30–34 vs. 35–37 y*.

*c: <30 y vs. 35–37 y*.

*d: 38–40 y vs. 41–43 y*.

*e: 41–43 y vs. 44–50 y*.

*: *P < 0.05*.

Singleton birth neonatal outcomes following the first freeze-all FET cycle are given in Table 3. Risk of PTD, very PTD, LBW, very LBW, term LBW, preterm LBW, and macrosomia did not change significantly as a function of maternal age.

**Table 3:** Neonatal outcomes of live born singletons.

**Characteristics**	**<30 y (*N* = 430)**	**30–34 y (*N* = 578)**	**35–37 (*N* = 270)**	**38–40 (*N* = 116)**	**41–43 (*N* = 47)**	**44–50 (*N* = 9)**	***P***
VLBW (<1,500 g)	4 (1.04%)	5 (0.79%)	2 (0.86%)	1 (1.01%)	1 (2.13%)	0 (0%)	a, b, c, d, e
LBW (<2,500 g)	15 (3.49%)	24 (4.07%)	14 (5.19%)	7 (6.04%)	3 (6.38%)	0 (0%)	a, b, c, d, e
Term LBW	1 (0.23%)	4 (0.69%)	2 (0.74%)	1 (0.86%)	1 (2.13%)	0 (0%)	a, b, c, d, e
Preterm LBW	14 (3.26%)	19 (3.31%)	14 (5.19%)	6 (5.17%)	2 (4.26%)	0 (0%)	a, b, c, d, e
Macrosomia (>4,000 g)	23 (5.35%)	35 (6.06%)	17 (6.29%)	5 (4.31%)	2 (4.26%)	0 (0%)	a, b, c, d, e
Birth weight (g)	3,389.53 ± 497.38	3,372.53 ± 513.37	3,343.67 ± 506.93	3,339.83 ± 519.52	3,334.54 ± 503.94	3,289.29 ± 521.69	a, b, c, d, e
Length at birth (cm)	50.33 ± 2.59	50.29 ± 2.75	50.21 ± 3.13	49.63 ± 3.25	49.38 ± 3.21	49.31 ± 3.29	a, b, c, d, e
**Gestation weeks at delivery (weeks)**							
Very PTD (<32 weeks)	5 (1.16%)	7 (1.21%)	3 (1.11%)	1 (0.86%)	1 (2.13%)	0 (0%)	a, b, c, d, e
PTD (<37 weeks)	28 (6.51%)	38 (6.57%)	18 (6.67%)	8 (6.89%)	4 (8.51%)	0 (0%)	a, b, c, d, e
**Child's sex, no**.							
Male	207 (48.14%)	282 (48.79%)	134 (49.63%)	59 (50.86%)	22 (46.81%)	5 (55.56%)	a, b, c, d, e
Female	223 (51.86%)	296 (51.21%)	136 (50.37%)	57 (49.14%)	25 (53.19%)	4 (44.44%)	
**Mode of delivery**							
Vaginal	167 (38.84%)	183 (31.66%)	73 (27.04%)	23 (19.83%)	8 (17.02%)	1 (11.11%)	a^*^, b^*^, c^*^, d^*^, e^*^
Cesarean section	263 (61.16%)	395 (68.34%)	197 (72.96%)	93 (80.17%)	39 (83.92%)	8 (88.89%)	

*a:<30 y vs. 30–34 y*.

*b: 30–34 vs. 35–37 y*.

*c: <30 y vs. 35–37 y*.

*d: 38–40 y vs. 41–43 y*.

*e: 41–43 y vs. 44–50 y*.

*: *P < 0.05*.

### Logistic Regression Analysis

A logistic regression analysis suggested that clinical pregnancy and live birth rates declined significantly with increasing maternal age, whereas miscarriage rates rose significantly as a function of maternal age. There were no significant differences in the rates of adverse neonatal outcomes as a function of maternal age with or without adjustment of the analysis results (Table 4).

**Table 4:** Crude and AORs for pregnancy and adverse neonatal outcomes in singleton births.

**Characteristics**	**<30 y**	**30–34 y**	**35–37**	**38–40**	**41–43**	**44–50**
**Clinical pregnancy**						
Crude OR(95% CI)	Reference	0.80 (0.69–0.92)	0.74 (0.62–0.88)	0.47 (0.38–0.58)	0.27 (0.20–0.35)	0.11 (0.08–0.17)
aOR(95% CI)	Reference	0.84 (0.73–0.97)	0.82 (0.68–0.99)	0.52 (0.42–0.65)	0.30 (0.22–0.40)	0.13 (0.09–0.20)
**Miscarriage**						
Crude OR(95% CI)	Reference	1.50 (1.07–2.12)	1.58 (1.05–2.38)	4.78 (3.15–7.24)	6.63 (3.86–11.37)	20.88 (9.19–47.44)
aOR(95% CI)	Reference	1.49 (1.06–2.11)	1.55 (1.01–2.29)	4.29 (2.78–6.62)	5.92 (3.38–10.39)	17.99 (7.77–41.66)
**Ectopic pregnancy**						
Crude OR(95% CI)	Reference	1.00 (0.57–1.75)	0.56 (0.24–1.33)	1.15 (0.48–2.73)	0.82 (0.19–3.55)	NA
aOR(95% CI)	Reference	1.01 (0.57–1.80)	0.57 (0.24–1.38)	1.18 (0.48–2.91)	0.70 (0.16–3.15)	NA
**Live birth**						
Crude OR(95% CI)	Reference	0.75 (0.65–0.86)	0.68 (0.57–0.82)	0.33 (0.26–0.41)	0.18 (0.13–0.25)	0.04 (0.02–0.08)
aOR(95% CI)	Reference	0.78 (0.67–0.90)	0.76 (0.63–0.91)	0.37 (0.30–0.47)	0.20 (0.14–0.28)	0.05 (0.03–0.10)
**VLBW (<1,500 g)**						
Crude OR(95% CI)	Reference	1.05 (0.80–1.38)	0.85 (0.61–1.17)	0.80 (0.52–1.23)	1.48 (0.73–3.00)	NA
aOR(95% CI)	Reference	1.01 (0.77–1.33)	0.75 (0.54–1.05)	0.64 (0.41–1.01)	1.20 (0.58–2.47)	NA
**LBW (<2,500 g)**						
Crude OR(95% CI)	Reference	0.95 (0.53–1.71)	1.23 (0.63–2.40)	1.06 (0.42–2.70)	0.42 (0.06–3.22)	NA
aOR(95% CI)	Reference	1.00 (0.56–1.81)	1.26 (0.63–2.51)	1.15 (0.44–3.02)	0.47 (0.06–3.66)	NA
**Macrosomia(>4,000 g)**						
Crude OR(95% CI)	Reference	1.34 (0.80–2.22)	0.75 (0.37–1.53)	1.04 (0.44–2.47)	0.72 (0.17–3.14)	NA
aOR(95% CI)	Reference	1.35 (0.81–2.26)	0.70 (0.34–1.45)	0.87 (0.35–2.14)	0.68 (0.15–3.05)	NA
**Very PTD (<32 weeks)**						
Crude OR(95% CI)	Reference	1.24 (0.45–3.45)	0.53 (0.11–2.63)	0.78 (0.45–1.38)	1.03 (0.73–1.43)	NA
aOR(95% CI)	Reference	1.27 (0.47–3.48)	0.51 (0.09–2.61)	0.75 (0.45–1.29)	1.05 (0.74–1.47)	NA
**PTD (<37 weeks)**						
Crude OR(95% CI)	Reference	1.10 (0.68–1.78)	1.18 (0.67–2.10)	0.86 (0.37–2.00)	0.29 (0.04–2.18)	NA
aOR(95% CI)	Reference	1.12 (0.69–1.81)	1.16 (0.65–2.09)	0.85 (0.36–2.04)	0.28 (0.04–2.10)	NA

*Analyses were adjusted for maternal body mass index, gravidity, parity, duration of infertility (continuous), infertility diagnosis, fertilization method, previous FET attempts, fertilization method, FET endometrial preparation, embryo developmental stage at transfer and number of embryos transferred. CI, confidence interval; OR, odds ratio; aOR, adjusted odds ratios. NA, not available*.

## Discussion

This retrospective analysis of 4,958 women being treated for infertility, leading to 1,450 singleton live births, provides novel evidence that clinical pregnancy and live birth rates decrease significantly with rising maternal age in the context of a freeze-all FET strategy, whereas miscarriage rates rise with increasing maternal age. There was no observed relationship between maternal age and any monitored adverse neonatal outcomes.

The freeze-all strategy has been increasingly popular over recent years, with some studies having observed markedly elevated implantation and ongoing pregnancy rates for this freeze-all approach relative to fresh transfer cycles, with increasing benefits for the freeze-all strategy observed with more advanced maternal age (21). However, while several studies have assessed how maternal age impacts implantation rates and other relevant outcomes in the context of FET cycles (5, 22), significantly less research is available with a focus specifically on the freeze-all transfer method. This is an important distinction to draw, given that FET analyses may incorporate transferred supernumerary embryos that remained following the transfer of better-quality embryos in previous cycles, making it important that freeze-all cycles be specifically investigated.

Our results suggest that despite comparable rates of transfer of morphologically good embryos in all study age groups, there was an age-dependent reduction in LBR, with rates of 51.15, 43.86, 41.64, 25.67, and 15.58% in women aged <30, 30–34, 35–37, 38–40, and 41–43 years, respectively, with a nearly 70% decline in women aged 44–50 years based on a freeze-all approach. These values were higher than LBR values for previously reported studies of conventional FET. One retrospective study of outcomes for 416 women that underwent transfer of frozen-thawed cleavage-stage embryos with a natural or CC/letrozole-based minimal stimulation protocol observed LBRs of 30.8, 37.5, 24, 7.8, 0% in women aged ≤29, 30–34, 35–39,40–44, and ≥45 years, respectively (22). Another prospective analysis of 1213 FET cycles conducted from 2000–2013 observed LBRs for women aged 38–39, 40–41, 42–43, and ≥44 years old of 14.1, 10.4, 3, and 0%, respectively (5). Our findings suggest that the freeze-all strategy may be a viable approach for women suffering from infertility regardless of age, with some hypothesizing that the freezing process may serve to filter out poor-quality embryos unable to survive the thawing process (23). Vitrification was routinely used for women in our study, while previous studies have used other approaches such as a slow freezing protocol (5). These differences in freezing strategies have the potential to lead to underestimation of actual LBRs, as differences in outcomes for embryo vitrification and slow freezing have been previously observed (24). Our study also differed significantly from previous studies with respect to a range of parameters including causes of infertility and protocols employed, potentially resulting in different observed results. As such, a large multicenter randomized controlled trial is essential in order to definitively establish how maternal age influences pregnancy outcomes in the context of a freeze-all FET strategy.

We did not detect any significant link between maternal age and risk of neonatal outcomes such as PTD, very PTD, LBW, very LBW, term LBW, preterm LBW, or macrosomia. This is in contrast to many studies identifying a positive correlation between maternal and age LBW/PTD risk in the context of spontaneous conception, even after adjusting for parental characteristics (20, 25–27). Other studies, however, have suggested that a more advanced maternal age is not associated with elevated LBW/PTD following adjustment for factors also shared by siblings (28). Relatively few studies have specifically assessed how maternal age affects neonatal outcomes in the context of ART (10). Wennberg et al. observed no significant increase in risk of PTD, very PTD, LBW, very LBW, SGA, or high birth weight (>4,500 g) following fresh embryo transfer for singleton births in mothers >35 years old, whereas spontaneous conceptions in comparably aged women were associated with elevated risks of PTD, very PTD, LBW, and SGA (10). Rates of PTD have been found to decrease with rising maternal age following fresh non-donor IVF cycle transfer according to a large study based upon the Society for Assisted Reproductive Technology Clinic Online Reporting System data (11). This is consistent with our findings, suggesting maternal age is not a strong determinant of neonatal outcomes in ART-derived pregnancies. All patients in the present study were implanted with good-quality embryos under more physiologically normal intrauterine conditions, thus potentially enhancing endometrial receptivity, early implantation, placentation, and fetal growth, thereby resulting in more favorable neonatal outcomes for FET than for fresh-embryo transfer.

This is the first study we are aware of to specifically examine how maternal age influences pregnancy and neonatal outcomes specifically in the context of a freeze-all FET approach. There are, however, several limitations to this study. Because of its retrospective nature, there may have been many age-associated differences in patient baseline characteristics across groups. We did carefully control for such characteristics where possible, focusing only on women undergoing their first embryo transfer cycle. However, there were differences in stimulation protocols, demographics, and endometrial preparation methods between groups. In addition, the 44–50 year old cohort of singleton live births was very small, limiting the power of statistical analyses of this group. Our study did, however, have many strengths, including its large cohort size and the fact that it was a single-institution study focused on a single year during which time consistent methodologies could be assured, guaranteeing no change in IVF procedures or laboratory conditions over the study period.

In summary, these findings indicate that a freeze-all strategy can mediate high rates of live birth per transfer cycle for women suffering from infertility, although rates of implantation, clinical pregnancy, multiple pregnancy, and live birth decline as maternal age rises, whereas miscarriage risk increases. We further found that there was no apparent association between maternal age and adverse neonatal outcomes in the context of pregnancies derived from a freeze-all FET approach. These findings are significant for both women of advanced reproductive age undergoing ART and for the clinical professionals responsible for their treatment.

## Data Availability Statement

The datasets generated for this study are available on request to the corresponding author.

## Ethics Statement

The studies involving human participants were reviewed and approved by The Ethics Committee (Institutional Review Board) of Shanghai Ninth People's Hospital. Written informed consent for participation was not required for this study in accordance with the national legislation and the institutional requirements.

## Author Contributions

JL, RC, and YW designed and performed the study, analyzed the data, and wrote and edited the manuscript. YK conceived and participated in the study design, evaluated the results and edited the manuscript. JH contributed to data collection and statistical analysis. QZ assisted in data acquisition. All authors have read and approved the final manuscript.

### Conflict of Interest

The authors declare that the research was conducted in the absence of any commercial or financial relationships that could be construed as a potential conflict of interest.
